# A catalyst for system change: a case study of child health network formation, evolution and sustainability in Canada

**DOI:** 10.1186/s12913-017-2018-5

**Published:** 2017-02-01

**Authors:** Charmaine McPherson, Jenny Ploeg, Nancy Edwards, Donna Ciliska, Wendy Sword

**Affiliations:** 1 School of Nursing, Faculty of Science, St. Francis Xavier University, Box 5000, Antigonish, Nova Scotia B2G 2W5 Canada; 2 School of Nursing, Faculty of Health Sciences, McMaster University, 1200 Main Street West, Hamilton, Ontario L8N 3Z5 Canada; 3 School of Nursing, Faculty of Health Sciences, University of Ottawa, 451 Smyth Road, Ottawa, Ontario KlH 8M5 Canada

**Keywords:** Health network, Case study methods, Child and youth health, Inter-organizational, Cross-sectoral, System responsiveness, Policy and politics, Interprofessional collaboration, Place-based approach, Collective impact approach

## Abstract

**Background:**

The purpose of this study was to examine key processes and supportive and inhibiting factors involved in the development, evolution, and sustainability of a child health network in rural Canada. This study contributes to a relatively new research agenda aimed at understanding inter-organizational and cross-sectoral health networks. These networks encourage collaboration focusing on complex issues impacting health – issues that individual agencies cannot effectively address alone. This paper presents an overview of the study findings.

**Methods:**

An explanatory qualitative case study approach examined the Network's 13-year lifespan. Data sources were documents and Network members, including regional and 71 provincial senior managers from 11 child and youth service sectors. Data were collected through 34 individual interviews and a review of 127 documents. Interview data were analyzed using framework analysis methods; Prior's approach guided document analysis.

**Results:**

Three themes related to network development, evolution and sustainability were identified: (a) Network relationships as system triggers, (b) Network-mediated system responsiveness, and (c) Network practice as political.

**Conclusions:**

Study findings have important implications for network organizational development, collaborative practice, interprofessional education, public policy, and public system responsiveness research. Findings suggest it is important to explicitly focus on relationships and multi-level socio-political contexts, such as supportive policy environments, in understanding health networks. The dynamic interplay among the Network members; central supportive and inhibiting factors; and micro-, meso-, and macro-organizational contexts was identified.

## Background

For the past two decades interdisciplinary, inter-organizational, cross-sectoral, and community-based collaborative partnerships, such as inter-organizational networks and health coalitions, have been proposed as key strategies to improve public health system performance, service access and coordination, and overall health outcomes [1–13]. We continue to see proliferation of health networks and continued discussion regarding their ‘fit’ in many jurisdictions, including Canada, the US, and the UK [14]. We also have seen recent emergence of global health networks to address issues such as tuberculosis, maternal mortality and newborn deaths in low- and middle-income countries [15]. Network researchers argue that inter-organizational collaboration through health networks is one of the most promising practice-based approaches in the public health field today [13, 16–18]. The health network approach holds particular promise to address complex and intractable issues of child and youth health that inherently involve many sectors, such as family violence, addictions, and family poverty. However, inter-organizational networks are a lot of work, are resource intensive, require shared leadership and some loss of control, and “should be considered as a policy instrument only when indicated (i.e., for complex endeavours where inter-organizational collaboration is a necessity)” [14]. The purpose of this paper is to report the results of a retrospective case study of the development of an inter-organizational child health network in Canada. Results of this study offer new guidance for child health practitioners and policy-makers as they consider the web of processes and key factors impacting the development, evolution and sustainability of their networks.

Collaborative partnerships are seen as a way to attain resources, share knowledge, and improve outcomes for complex and often socially determined issues that have roots in many sectors [16, 17, 19–23]. In an attempt to break down the traditional silos of service sectors, public systems have structurally embedded such partnership models by integrating networks into their core business processes and governing legislation [24, 25]. Multi-organizational partnership has been found to strengthen primary health care [26, 27] and collaborative teamwork for clinical management and research [28–32].

A systematic review examining the empirical research on the structure of networks of health professionals, with regard to the effectiveness and sustainability of networks, focused on quality of care and patient safety [33]. Findings showed that cohesive and collaborative health professional networks can facilitate the coordination of care and contribute to improving quality and safety of care. However, evidence about the way health networks develop and evolve is currently limited and fragmented [17, 33].

The use of networks for the child and youth population is important to consider because complex child and youth health concerns are often cross-sectoral in nature and solutions require the involvement of many stakeholders. Further, the promise of positive outcomes for prevention and early intervention strategies with the child and youth population is high since they are young. Collaborative partnerships across organizations under a networked umbrella are a way to address system complexity for children and youth [14, 20, 34–37].

### What are inter-organizational child health networks?

The literature informing inter-organizational health networks draws from several scholarship streams, such as public administration [38], business [39], and health promotion [17]. Short and colleagues [17] proposed the following definition of *networks*:

A network is a group of multiple entities which are tied together with some form of structural peer-to-peer interdependence and common interest. They jointly coordinate their activities without subordination and form relatively stable, flexible working relationships. A network is characterized by open-ended relationships and distributed tasks requiring input from several members. Networks typically help with knowledge translation and promote diffusion and sharing of information and resources. [p. 2]

The notion of horizontal coordination of services for children and youth has been an ongoing public policy concern [19, 40]. Our deepening understanding of the intersecting nature of many complex child and youth health problems, including balancing prevention and early intervention with acute care services, has made the issue of horizontal coordination even more pressing [41]. Governments have responded to these cross-cutting health problems with various policy reforms and innovations, including network governance [14, 38, 42]. Networks may “reach places that formal structures and hierarchies cannot” [14] (p. 15). Sørensen and Torfing [43] considered the current wave of new public governance reforms, noting that collaboration between relevant and affected actors from the public and private sector are perceived as the primary vehicle of public innovation, with governance networks as potential arenas for collaborative innovation. They suggest that the purpose of governance networks is to stimulate efficiency, effectiveness, and democratic legitimacy through innovation. Thus, governments may choose inter-organizational networks as a ‘tool’ or intervention strategy for government policy aims – as a means to tackle critical social and economic policy goals [14].

When considered as a type of governance structure, or way of organizing and governing to get messy, collaborative work done, the network form can be distinctly characterized by several aspects including: (a) a spirit of goodwill, (b) high levels of trust between parties, (c) norms of reciprocity and adaptability, (d) a sense of obligation among group members, and (e) embedded ties through strong and enduring relationships [44]. Several of these network characteristics are discussed in the social network and health literature [45, 46].

Since the late 1990s inter-organizational child health networks, which focus on the child and youth population, have gained some hold in Canada [47, 48] and other countries, such as Australia and the UK [14, 17]. Child health networks are formed when many child serving agencies informally come together under a single networked umbrella to work collectively on common goals. The network members retain their own organizational identities (e.g., as school boards, mental health services, child protection agencies), but they add the network affiliation as another layer – sitting collectively beyond the tangible organizational boundaries within which they traditionally work. Child health networks might be anchored to one or several of their member agencies to support day-to-day operations [34–36]. Child health networks may develop partnerships that span regional, provincial, or federal governmental boundaries. These complex partnerships are formalized within the child health network to support efforts concerning cross-cutting social issues that impact health, such as child poverty, family violence, and cross-sectoral and pan-governmental service planning and delivery. Child health networks may also work on more local or micro­issues, such as harmonized service entry points and inter-organizational service agreements and policies [34–37, 49].

### Child health networks in current context

There has been an increased focus on early childhood and the expansion of *place-based* approaches to outcome improvement [50–52].

Place-based approaches aim to address complex problems by focusing on the social and physical environment of a community and on better integrated and more accessible service systems, rather than focusing mainly on the problems faced by individuals. A place-based approach targets an entire community and aims to address issues that exist at the neighbourhood level, such as poor housing, social isolation…by using a community-engagement approach to address complex problems, a place-based approach seeks to make families and communities more engaged, connected and resilient [50].

The place-based approach encompasses aspects also inherent in inter-organizational child health networks, such as the multiple layers and influences on child health (i.e., system and problem complexity from a social determinants of health perspective) [19, 53, 54], and the value placed on prevention, early intervention, and community and citizen engagement [54–56].

There is no formal typology that differentiates health networks from coalitions in the health literature. Health coalitions are a similar organizational genre as networks [12], and so we consider this literature. While the formation of networks may be considered as a more formal government service coordination response to cross-cutting policy issues, coalitions developed primarily by stakeholders outside of government control have also emerged on a more informal basis. Consistent with definitions of networks, health coalitions are described as unions of people from multiple sectors that come together to collectively address a range of goals that are unattainable by a single sector or organization [12, 57–59]. One distinction is that the networks discussed in this paper are formed under the auspices of government while coalitions are often partnerships uniting stakeholders to monitor and advocate for government action or change. While the benefits of coalitions in public health have been widely accepted [1], the evidence of impact is weak [16, 60], and there is no guidance on long-term viability [61]. Although community-based programs are often evaluated to establish short-term effectiveness [7, 62], until recently, little attention has been paid to whether, how, or why programs and the associated partnerships, systems changes, and direct services sustain themselves in the community over the long-term [38, 42, 61, 63–65]. We do know that the context within which the partnership operates is of utmost importance in determining the factors affecting long-term sustainability [61]. For example, if a network operates within an unstable provincial government context with reactionary and shifting priorities due to the election cycle, then the long-term sustainability of the network as a policy intervention tool may be unstable as well.

In Canada we have seen coalition development that is provincial or national in scope that fulfills an advocacy function for children and youth, such as British Columbia’s First Call BC Child and Youth Advocacy Coalition [66, 67]. First Call is a non-partisan coalition of over 95 provincial and regional organizations united to advocate for children and youth in BC through public education, community mobilization, and public policy advocacy. In a liaison fashion, government departments or organizations may participate in the coalition without voting rights. The First Call coalition developed the Early Childhood Development Roundtable, bringing together early childhood advocates to monitor how public policy and investments are serving children. Examining the state of services and supports in their local communities and new developments in their fields of work, the roundtable has regular participation from officials from provincial, federal and some municipal governments. First Call [67] indicates that this facilitates dialogue and feedback to inform public policy development and to share government plans and intentions with members of the early childhood field. In this coalition the locus of control is clearly within the coalition itself, rather than being driven by the provincial government.

There has been tremendous growth in the inter-organizational health network literature over the past decade [14, 68]. There is preliminary evidence of process indicators and health outcomes that suggest the usefulness of some types of health networks [3, 5, 13, 14, 49, 68, 69]. However, there is limited empirical evidence or scholarly discussion to support inter-organizational child health network practice, particularly how to develop, evolve and sustain these networks [14]. Despite limited evidence, child health networks and similar collaborative partnerships continue to be developed in many jurisdictions because they are seen as innovative and hold much promise for strengthening health systems. At the same time, long-standing child health networks are being dismantled in some jurisdictions.

The purpose of this study was to examine the development, implementation and sustainability of an inter-organizational child health network. The case study was guided by the primary research questions: *What are the key processes related to network formation, evolution and sustainability? What supportive and inhibiting factors influence these processes?*

### Introduction to the case

The Network for Children and Youth of Eastern Nova Scotia (herein called the *Network*) was developed in 1994 under a provincial policy directive [70, 71].

#### Impetus for formation

The impetus for Network formation as a government strategy related to multiple contexts: clustered multiple youth suicides in a rural high school in eastern Nova Scotia, several years of inter-organizational planning around youth health service models, a longstanding concern over lack of inpatient and outpatient children's mental health service availability and accessibility, and a growing international policy recognition of the critical importance of the early childhood period in terms of lifelong health and well-being [72].

#### Mandate

The Network mandate as set out in the initial Terms of Reference [70, 71] was to “integrate services” across key child and youth services, such as inpatient mental health services, child welfare services and youth restorative justice and criminal justice programs. As the Network matured, the mandate evolved to a broader view of improving health and well-being outcomes by collaborating on issues that address the social determinants of health for the child and youth population in the region.

#### Leads

The four regional administrators of the provincial Departments of Health, Community Services, Education and Justice were charged with responding to the policy directive that outlined interdepartmental and inter-organizational collaboration to advance the child and youth health agenda in the eastern part of the province.

#### Network membership: inter-organizational and cross-sectoral partners

It was from this initial interdepartmental collaborative mandate that the Network developed, ultimately consisting of upwards of 48 child and youth serving organizations – government funders, government services, and NGOs. Senior managers, usually at the CEO or Director level, from child and youth serving organizations (e.g., regional school boards, child welfare agencies, regional health boards, youth criminal justice programs, family resource centres, government departments) came together under the Network umbrella to consider issues and strategic directions that they could collectively tackle. Despite the voluntary nature of Network membership, the uptake and participation rate was significant and longstanding.

#### Network staff

By 1996, the Network was permanently staffed with a full-time Executive Director and a full-time office manager. This staffing pattern was maintained over time. The staff was co-located with one of the four main Network co-leads as an administrative home.

#### Network governance

The Network was initially governed by a Steering Committee (four regional administrators of lead Departments and the Network Director), and an advisory body (representatives from other Network member agencies). Child, youth and family input was sought through existing channels within the member agencies and through specific pilot project representation.

## Methods

Explanatory case study approach [73] with theoretical propositions guided this study. Consistent with Yin’s approach to this method, propositions related to the substantive research questions were drawn from existing theory and empirical research on inter-organizational networks and health networks. Critical for this case study was the use of propositions to: (1) direct attention to particular concepts that should be examined within the scope of the study, and (2) support study feasibility by focussing the relevant evidence in data collection and analysis [73, 74]. The propositions are theoretical statements taken from existing literature and thus provide the theoretical grounding to guide the study. Table 1 summarizes the study propositions.

**Table 1:** Study theoretical propositions

Proposition 1	Child health network organizations foster the development of embedded ties and a sense of social connectedness among network members. Formation of embedded ties is reflective of “the duality of structure and the recursiveness of social praxis, thus attending to social embeddedness and co-evolutionary processes in network life” [64]. This refers to the close connection between the developing structure of the network and the practice side of network life. Sydow [64] suggests that the process of developing social connections within the network is key to its evolution.
Proposition 2	Underlying the development of embedded ties in child health networks are normative standards of reciprocity and trustworthiness that are traditionally fundamental to network forms of organization. Each member of the child health network feels a sense of obligation to the other party or parties rather than a desire to take advantage of any trust that may have been established [118].
Proposition 3	It is crucial that network members purposefully maintain a contextual and systemic orientation as members navigate the internal and external historical, cultural, political, and economic influences on network formation, evolution, and sustainability [97–99, 119]. This means that the contexts and the public systems surrounding networks matter or have an impact on them. Particular contexts are worth paying attention to including historical, cultural, political and economic contexts.
Proposition 4	There is a macro-micro level tension created by the external historical, political, and social institutional forces and the more local internal micro-level organizational forces that are developing and evolving [120, 121]. This proposition seeks to expand our understanding of proposition 3 to particularly consider how the macro-level forces that originate outside the network may impact the internal network experience. For example, a history of poor communication between child and adolescent mental health services and the local schools (historical macro-micro level tension) gets brought into the developing child health network and may impact how these two sectors work with each other on initiatives within the developing network, perhaps even negatively, affecting the network organization success.
Proposition 5	Historical social and political institutional forces encourage growing formalization and centralization of the network, emulating traditional public service sectors [102]. This means that, even though network organizations are designed as a counter to traditional public service sectors to deal with their known constraints (e.g., hierarchy, slowed and siloed decision-making, turf protection), there is still a natural tendency for network members to lean towards developing the network organization in the traditional ways in which they have been educated and socialized. There are also political forces that drive a network towards traditional public service models, such as expecting a network to fit into standard governmental reporting and daily communication patterns. In this way, the networks end up ‘emulating’ or patterning themselves after traditional public service sectors.

A child health network from eastern Canada (the *Network*) was selected as a unique organizational case. At the time of this study, it was regarded as a pioneer and leader in the field, and had been the longest-standing child health network in Canada [71, 75]. This case was bound by time (June 1994 to June 2007); place (regional/provincial/national geographical and political boundaries); organizational definition (outlined in Network Terms of Reference) [71]; and context, with a particular focus on child and youth health and human services.

Data sources included people and documents. Purposeful sampling with maximum variation and predefined criteria (see Table 2) was used to seek sample diversity and breadth across Network members, staff, and external partners. An emailed letter and information sessions were used to introduce the project and recruit participants. Interview data were collected between May and December 2006. In-depth individual semi-structured interviews lasting approximately 1.5 h were digitally recorded and transcribed. The interview guide consisted of 20 questions, such as: What processes supported early Network formation? Describe how your role in your home organization may or may not have affected Network formation? What challenges affected Network organizational progress? What factors challenged Network sustainability? This guide changed over the course of the scheduled interviews to reflect the developing themes as data collection and analysis proceeded.

**Table 2:** Socio-demographic characteristics of interview participants (*n* = 34)

Characteristic (sampling criteria)	*n*	%
Gender:		
Female	22	64.7
Male	12	35.3
Age (mean = 49 years):		
< 40	3	8.8
40–50	16	47.1
> 50	15	44.1
Sector:		
Community Services^a^	8	23.5
Health	8	23.5
Education	5	14.7
Other^b^	13	38.2
Location:		
Within Network Region	30	88.2
Outside Network Region	4	11.8
Years in Profession:		
< 20	4	11.8
20–30	14	41.2
> 30	6	17.6
Length of Network Membership (years):		
< 5	4	11.8
5–7	9	26.5
8–10	11	32.4
> 10	10	29.4

Actual numbers based on sampling criteria categories (for example, the number of participants and their actual number of practice years >10) cannot be disclosed in an effort to protect participants’ identities. ^a^Community Services refers to the Nova Scotia Department of Community Services. This includes affiliate social services agencies that receive core funding from the Department of Community Services. ^b^Other sector category includes 8 different sectors that are clustered in this generic category (numbers per sector too small to report)

Spencer, Ritchie, and O'Connor's [76, 77] Framework Analysis guided analysis of the interview data. Framework analysis was developed in the context of applied policy research [78], and is increasingly used in applied health research in combination with case study methods [79, 80]. Conceptual scaffolding, a particular method within framework analysis, and its five iterative stages and processes was followed: (a) familiarization, (b) identifying a thematic framework, (c) indexing, (d) charting, and (e) mapping and interpretation. Interview data were indexed and coded using the NVivo7® software, and several software functions were used to support analysis, including memoing, annotations, and matrices. CMP developed codes under the thematic framework, which were verified by JP. All co-authors reviewed the developing codes and themes.

A variety of Network and contextual documents were purposefully sampled using pre-defined criteria (see Table 3). Documents were collected through an informant process whereby key people associated with the Network were asked to identify documents that related to the study questions [73, 81]. Documents were retrieved between May 2006 and May 2007 and logged. These documents were analyzed within their social setting as situated products to trace patterns of social exchange and the social networks behind them [81]. Particular attention was paid to: (a) content, not their fixed meaning but a situated or referenced meaning; (b) how they were produced; and (c) how they functioned or their use. Each document was systematically analyzed using a framework that included questions such as: Whose perspective was reflected in the document? How did the document function in terms of Network formation events and processes?

**Table 3:** Characteristics of study documents (*n* = 127)

Document Type (sampling criteria)	Examples	*n*	%
Policy & Planning	• Eastern Region Child & Youth Services Project Protocol • Network Strategic Plan • Nunn Commission Report • Reports to provincial committees	39	30.7
Executive Meeting Minutes	• Project Steering Committee meeting agendas and minutes • Network Executive Committee meeting agendas and minutes	29	22.8
Council Meeting Minutes	• Network Executive Committee meeting agendas and minutes • Project Advisory Committee Reports, meeting agendas, minutes	9	7.1
Annual General Meeting (AGM) Materials	• AGM Programs • AGM minutes • Director’s Annual Reports • Chairperson’s Annual Report • Annual Financial Statements	25	19.7
Special Project Reports	• Pilot project proposals • Project brochures • Pilot project evaluation reports • Network website	11	8.7
Other Communication Materials	• Network website • Network newsletters • Emails sent through listserv	14	11.0

Lincoln and Guba's [82] guidelines for establishing rigour or trustworthiness (i.e., credibility, transferability, and dependability) in qualitative research were blended with Morse and colleagues’ [83] recommendations for ensuring active verification strategies. Consistent with a case study approach, a chain of evidence was systematically established during data analysis and interpretation, including consistent testing against the study propositions. There was a deliberate focus on divergent patterns, negative instances, alternative themes, and rival explanations [73].

## Results

A sample of 34 participants (Table 2) and 127 documents (Table 3) was achieved.

Three themes and their associated subthemes were identified (Table 4) and are described below, with supporting quotes identified by participant number.

**Table 4:** Study themes and subthemes

Theme 1: Network relationships as system triggers	Theme 2: Network-mediated system responsiveness	Theme 3: Network practice as political
• Trust • Interdependence • Positive peer influence • Power imbalances	• Network staff responding to members • Network members and their organizations responding to each other • Network responding as a collective	• Senior management engagement • Organizational legitimacy • Provincial political factors

### Theme 1: Network relationships as system triggers

New professional relationships that developed and evolved under the auspices of the Network were seen as system triggers; these relationships *triggered* change within the Network and within the Network members’ work-life systems. Participants consistently referred to relationships among Network members and staff as integral to the Network organization and its mandate, as this interview excerpt highlights:

We try to compartmentalize things – that's how we run our day-to-day business. But in the Network we’re trying to come back to this holistic approach… I think the Network works on personal working relationships. The Network is not a command and control model, and it shouldn't be. (P13)

Network relationships were identified as *interdependent* and *synergistic*. Many Network activities took place through interdependent relationships that were initially sparked and then maintained through Network engagement. The interdependency among Network relationships connected members in new and dynamic ways.

We identified three specific facilitating factors within this relationship theme: trust, interdependence, and positive peer influence, as well as one inhibiting factor – power imbalances.

#### Trust

Establishing and relying on trust-based Network relationships was critical, and was seen as a benefit of Network participation.

I really believe that having the common table and establishing trust and good communication is perhaps the biggest benefit that you can have in the Network …And at the end of the day, that's more powerful than policy, guidelines or regulations. (P1)

The whole concept of the Network from the get-go was so foreign to people, they weren't used to that – everybody coming together, partnering, collaborating, the integrated work…I think all that falls back onto developing the relationships and the level of trust that was created and that evolved over time. (P29)

Growing trust *triggered* further successful collaborative efforts:

…not everything becomes a Network initiative, but you get little bilaterals and trilaterals outside of the Network happening simply because those people [Network members] were there at the same time and they got talking to one another. They knew, trusted, and respected each other from their Network partnerships. (P10)

#### Interdependence

Close, reciprocal and interdependent relationships among Network members supported the development of new cross-sectoral partnerships outside the child and youth population. For example, the relationships established under the child Network further supported the development of a strategic plan across the same agencies for the continuing care sector. This interdependency occurred within a broader system’s context of cost-cutting and tremendous in-fighting for dwindling provincial funding. This was a time when public service managers tended to be ‘digging their heels in even deeper’ in their own familiar organizational silos and ways of working. However, for Network members, a heightened sense of reciprocity or interdependence for a collective purpose was created:

With the Network, it's changed our way of thinking. It's no longer 'what can we as a department do?' or 'what can the [another sector] do?’ when there’s a problem. It's 'what can we *all* do? How can we come to the table and solve this so everybody can contribute to problem solving rather than working independently?' Before the Network, we saw only one part of the issue. Within the Network, you see the whole picture. (P6)

Unexpected spin-offs were sparked by these interdependencies and collective commitment. The success of the Network resulted in the development of considerable organizational legitimacy among regional and provincial partners, which led to opportunities for further partnerships and initiatives. For example, the Network was asked to support the planning for early childhood intervention services expansion in the region. This was a positive impact because historically a central government department would likely have done this planning. A second example is when the Network members asked that the Network facilitate a collaborative process for the development of an inter-agency referral protocol for child and adolescent mental health services. These examples demonstrate how opportunities to work together on other issues arose from, or were triggered by, the highly interdependent nature of the Network relationships, as described by this participant:

There was more opportunity to develop creative partnerships because we were in regular contact around child and youth issues. It became the norm…you didn’t proceed with big change without checking in with your Network partners [from other organizations and sectors] because changes affect all of us…but this often lead to other opportunities to do some important work [described partnering around child and adolescent service waiting lists and development of interagency referral protocols]. (P10)

#### Positive peer influence

The Network relationships were described as providing positive peer influence among organizations represented by Network members. These peer influences were seen in strategic service and policy decision-making:

…if you’re thinking ‘my only reporting is to my department, my manager, my division in head office,’ then you cannot honestly do intersectoral work…I have to allow myself to be influenced by my peers… my decision-making is not uniquely what my higher-ups would say, it's also influenced by my [Network] colleagues on the side… (P6)

These relationships also stimulated creative and innovative solutions to complex child and youth inter-organizational service issues. For example, the development of an inter-organizational and cross-sectoral strategic plan to address child poverty was stimulated by the positive peer influence that some community-based Network partners had on more traditional governmental members. They influenced their colleagues and fostered the collective belief and vision that all had a role to play in addressing child poverty. As Network members became more familiar with each other’s organizations, they moved more quickly to issue planning and resolution and developed a new vision of what was possible. This was the ultimate expression of Network peer influence:

You reach the level of understanding and see the cross-sectoral implications of your own decisions …you could see those improve over the first couple of years [of the Network] …then you started seeing the vision and imagination – what we can do together – you think about this…you consider the impact on others and their agencies …we have the same clients. (P4)

#### Power imbalances

Interview and document data (e.g., Policy & Planning documents, May 2003) provided many examples of Network members from the health sector negatively using their power base and privilege, especially early in Network formation. For example, some Network members from the health sector expected more decision-making power or expected to be seen as the expert with the final ‘say’ on an issue because they were able to dedicate more money than other sectors to the Network budget. Senior participants linked this negative power use to an external cultural influence of traditional departmental sectors, which had notable differences in provincial budget allocations and staff disciplinary mixes. This situation created a power imbalance among Network members that was often at the root of relationship conflict within the Network. The power imbalance sometimes alienated colleagues from other sectors, and negatively influenced some committee work, strategic planning exercises, and progress on special projects. One participant connected the power dynamics with health professional/health sector cultures and the Network interdisciplinary and cross-sectoral context:

Some professional groups are more prone to using power dynamics than others…Of the people that I deal with through the Network, I would say that health professionals are the most controlling. As a group they tend to be more rigid in terms of roles than some of the other departments. Only one department has a two million dollar budget…When you're dealing with Health you are always sleeping with the elephant,^1^ it's their culture. (P3)

### Theme Il: Network-mediated system responsiveness

The Network served as a mediator in enhancing *system and organizational responsiveness*, which was described by participants as the ability to act quickly and appropriately with respect to system and organizational needs. We identified three distinctive ways in which Network-mediated responsiveness was realized: (1) Network staff responding to members, (2) Network members and their organizations responding to each other, and (3) Network responding as a collective (see Table 4).

Network staff provided support for Network members, enabling rapid action on priorities and a level of responsiveness that could not have been provided by individual organizational members. This statement from a participant demonstrates this responsiveness:

I go to a meeting and somebody says, 'Well, to do this policy well, we need to have justice and the police and transition houses at the table'. I simply call [name, Network staff] to set up the meeting because I know that planning vehicle exists for us. (P6)

As individual Network members and their home/member organizations learned about each other’s capacities, mandates, strengths and networks, they began to be more **responsive to each other and to their respective organizations**. Members’ positive experiences in working together and responding to each other within the Network created a synergy that carried over into contexts beyond the Network. For example, some Department of Community Services developed deeper working relationships with public health staff through child Network initiatives. This opened up communication and heightened collaboration pathways when the same staff were faced with working together to support other populations. This responsiveness to each other and to other organizations is identified in this interview quote:

The Network has opened the door with respect to other challenges. So, you'd have a senior client who's on income assistance that may have medical problems – it's much easier and quicker and effective for the worker to call Public Health directly now because they've connected with them through initiatives of the Network. (P16)

As the Network matured, and members became more deeply connected relationally, they were able to respond to external demands as **a collective**. They became more adept at creatively adapting and responding to internal/external contexts and demands. For example, when a local high school drug problem was identified by the school (an issue initially outside of the Network), the Network members were able to quickly collaborate to collectively develop a coordinated inter-organizational and cross-sectoral response. As a crucial supportive factor, system and organizational level responsiveness became the chief Network function, as identified by participants. Adaptability and nimbleness far beyond capacities of the traditional public service system were cited as examples of this collective responsiveness, which was deemed especially important for cross-sectoral issues:

The Network is a set of levers – you create an opportunity to bring people together and within minutes you're rolling up your sleeves and talking about how we're going to tackle mutual problems and issues. (P13)

I do think the Network members have the ability to react and shift direction if they need to without a whole bunch of problems that would go along with another government department going down that road. (P18)

Documents revealed that the Network continuously scanned its environment for opportunities to enhance their efforts and for issues that might undermine their mandate. Document data (Policy & Planning documents, March 2000; June 2007) revealed how the Network was able to positively respond to planning demands placed upon it by outside agents. For example, the provincial government and community partners requested that the Network lead a regional project focused on service enhancement for children diagnosed with Autism Spectrum Disorders (ASD). This was an emerging service area for the province that was also highly contentious and political involving advocacy by several interest groups. The Network was able to nimbly develop inter-organizational and cross-sectoral planning groups and engage with key service, community and user stakeholders to collaboratively examine the service needs, develop a program proposal, and then facilitate service development for children diagnosed with ASD and their families.

### Theme III: Network practice as political

The political aspect of Network practice was ongoing and involved individuals, institutions, and government at micro-, meso-, and macro-level contexts. Political practice concerned deliberate (Network-related) political actions in Network members’ home organizations as well as collective action concerning provincial governmental policies and politics. Both required an interface with processes and opportunities that were, at times, unpredictable, dynamic, and chaotic. We identified three subthemes: senior manager engagement, organizational legitimacy, and provincial political factors (see Table 4).

The engagement of **senior managers** from Network member agencies was a crucial supportive political factor that was especially critical during early Network formation. This engagement provided a senior administrative show of support for the initiative:

It doesn't matter whether it's regional or provincial, if you don't have the right people at the table, you're not going anywhere. [For the Network] you have to have your key regional decision-makers [on board] who are able to commit both fiscal and human resources to the endeavor in some way, otherwise it isn't going to work. (P17)

The Network’s **organizational legitimacy** and credibility gradually developed over time, and was identified as a key factor that supported Network political practice. Several of the aforementioned examples demonstrate how legitimacy was evident at multiple levels and with multiple stakeholders (i.e., internally to Network, and within member organizations, government, and community). For example, the Network was asked to manage several projects that required inter-organizational and cross-sectoral planning and political awareness, such as the early childhood interventions services expansion and the ASD service planning and implementation. These project management requests demonstrated that the Network had established service planning legitimacy with the government. The Network would not have been entrusted with this high priority work by the government if it had not been seen as a legitimate organization. Network organizational legitimacy developed over time and was considered a desirable outcome and a possible predictive indicator of organizational stability and sustainability.

We identified **provincial political factors** as a chief influence of Network development, evolution and sustainability. Participants overwhelmingly pointed to the Network mandate flowing from provincial policy as a very positive and powerful **catalyst** for early Network formation. This mandate (see Table 2 for case study description) set the stage for people to engage with one another to effect system change:

If that had not been mandated, then the Network wouldn't exist; you know these networks don't just spawn themselves. (P1)

As the Network evolved, it was again provincial policy that supported its evolution by providing **core operational funding** and **pilot project funding**. For example, beyond annual core funding, project funding was awarded to the Network for development of four school-based inter-organizational youth health centres in the region. This major planning project involved development of a youth health centre agenda among local and provincial stakeholders, including youth and parents, and 3 years of facilitated planning to realize the services. These pilot projects offered a provincially sanctioned focus, and the fact that the provincial government trusted the Network with additional projects served to further reinforce Network organizational legitimacy. The Network established deeper links with the provincial stakeholders, such as the Treasury Board, especially as costly long-term service planning was underway:

It's really important to engage not only the senior management civil servants from service delivery, but more and more l recognize that you need to engage Treasury and Policy Board…there need to be clear lines of communication and linkages through to your policy people for the Network. (P31)

Although Network relations with provincial staff and elected officials were generally described as positive and supportive, at times provincial staff tried to **control the Network strategic agenda** and micromanaged key shared projects (e.g., the ASD service project). One participant recounted the power of the provincial Department of Health staff in decision-making over local Network practice:

That project was a major issue that showed if the Department of Health takes a certain line – ‘this is what the rules are’ – then it can really impact how you get to play on the ground with your partners. That was probably the worst example of where that [provincial political micromanagement] really caused a lot of problems. (P6)

Periodically there was a **destabilizing effect of provincial government politics** on the Network, such as during electoral cycles. Government support wavered as Network activities became more complex and politically active, raising legislative and accountability issues. The Network functioned in a ‘grey zone’ between formalized provincial and regional governmental and community-based structures and organizations. Occasionally there was not great clarity in terms of the true level of political support for the Network way of working. For example, documents (e.g., Policy & Planning documents, October 1999; February 2006) indicated that the provincial government did not always share the same vision of their role as the Network members. The following quote exemplifies this issue:

It is really important to build the linkages across government departments in terms of a framework for joint policy development or impact assessment of policy to be supportive of regional [child health] networks. The provincial government needs to start role modeling their vision at that level; there is a joint growth that needs to happen for each to understand what the other is doing. (P14)

## Discussion

Consistent with Yin’s explanatory case study approach [73], this discussion revisits the initial theoretical propositions in developing a chain of evidence supporting the study findings. We identified three essential areas to be considered by leaders and practitioners in contemporary inter-organizational health network practice and policy. Although this study was completed in 2008, these findings remain relevant and valuable today, especially when examined through current collaborative trend lenses such as place-based [52] and collective impact [84] approaches to action for optimal child health outcomes.

The first major study contribution is a deeper understanding of the interdependent nature and synergistic impact of key aspects of network relationships. Consistent with our study findings, others have discussed the role of relationship building in collaborative health networks [7, 26, 85, 86] and successful knowledge translation. Supporting study proposition 1 (see Table 1), our study findings expand this understanding by identifying the interdependent and synergistic nature of key relational factors. These issues have received little attention within the inter-organizational health network literature, although these concepts arise in discussions regarding social networks and health issues [45, 46, 87], place-based approaches to collective action for children [50–52], and collective impact in large scale social change [84, 88–90]. The same concepts have been widely discussed in the public administration and business network literature [91–94]. Sydow and Windeler [95] talked about the formation of embedded ties as reflective of “the duality of structure and the recursiveness of social praxis…thus attending to social embeddedness and co-evolutionary processes in network life” (p. 265). ‘Recursiveness’ simply means that there is a rule or procedure that repeats on itself. In network practice, this is the embedded iterative feedback loop that provides information to inform change, strengthen relationships and create synergy. This in itself suggests some interdependency and that the process of developing social connections within the network is key to its evolution. We identified a close evolutionary connection between the developing structure of the network and the shifting day-to-day network practice. Our findings indicate that there is a sense of recursiveness in network practice that, in turn, influences what the network does and produces, and how it is structured.

The notion of relationships as a system trigger – connecting the relational aspects to enhanced system performance – was prominent. Current evidence in health literature has yet to link the synergy that is created by network-mediated interdependent relational factors to enhanced system activity and processes, although the business literature links relational networks to strategic advantage [96]. In 2011 Kania and Kramer [84] introduced the concept of *collective impact* in their analysis of the public school sector in the US. They argued that large scale social change requires broad cross-sector coordination, yet the public social sector primarily remains focused on the isolated interventions of individual organizations. Proposing a framework for collaborative work, they suggested that collective impact is different from traditional collaboration in that it is inclusive of a centralized infrastructure, a dedicated staff, and a structured process that leads to a common agenda, shared measurement, continuous communication and mutually reinforcing activities among all participants [84]. These key aspects of collective impact were present in the studied child health network. The notion of reinforcing activities parallels the idea of the recursiveness of the relationships as a trigger for system change. Further, the study data indicated that processes such as collective agenda setting, Network staff and planning infrastructure support (including provincial core and project funding), and clear communications are key processes and tangible resources to support child health networks in collective impact work. These findings expand our initial study theoretical proposition 2 (see Table 1) concerning normative standards of reciprocity and trustworthiness as traditional elements of network organizations. Our findings suggest that particular network factors (i.e., trust, interdependence, positive peer influence) form necessary conditions that extend Network-mediated activities and processes at a broader systems level. This new proposition deserves further investigation to examine the potential link between necessary conditions and sustained and impactful collaborative action.

The second major study contribution is an explanation of the multi-layered political nature of health networks. Key findings focused on the daily and ongoing political nature of health network practice, involving people, institutions, and government at micro-, meso-, and macro-level contexts. There was a dynamic relationship between the Network and its members’ multi-level contexts. Senior manager engagement, organizational legitimacy, and several provincial political factors (i.e., government as catalyst, operational and project funding, agenda controls, and destabilizing effect of provincial politics) influenced these relationships. As we posited (proposition 3, see Table 1), Network members needed to purposefully maintain a contextual and systemic orientation as they navigated the internal and external political influences on the network [61, 97–99].

Senior leaders used their own role legitimacy, reputations, and public systems knowledge to support the Network and develop its organizational legitimacy. These findings are important in public service contexts where intensive inter-organizational and collaborative work is often delegated to less senior staff. This is the first known health network study to clearly focus on the synergy that exists between senior management engagement and the Network organization’s multi-level political contexts.

Although the network form of organization is an innovative and trendy model with much potential, the Network functioned in a ‘grey zone’ between formalized provincial and regional governmental and community-based structures and organizations. The situation is similar with place-based and collective impact approaches, although the latter purposefully seeks engagement from a core group of ‘important’ actors – including influential heads of key organizations – who are able to abandon individual agendas in favour of a collective approach [84], as did the child health network in this study. Perhaps this organizational placement that is tethered to the public system is part of establishing organizational legitimacy and of engaging key organizational power brokers to buy into the collective change model. Place-based approaches and collective impact assessments/evaluation also steer away from the individualistic perspective and aim to address complex problems by focusing on the social and physical environment of a community and on better integrated and more accessible service systems. A place-based approach uses community engagement to address issues at a neighbourhood level, seeking to make families and communities more engaged, connected and resilient [52]. Regardless of the model, key processes and resources embedded within and cognizant of their historical, social, political and economic contexts and with leadership engagement remain foundational to collaborative change for children’s health outcomes.

Our finding regarding the destabilizing effect of provincial government politics also contributes to the child health network evidence base; we know that this stability and clarity is crucial for good public system functioning [100]. But a chief critique of organizational networks is the expectation that outcomes and processes will still be in line with traditional ways of working [101]. Our findings draw attention to the notion that, although governments may be using more collaborative arrangements, government managers' lack of understanding of what it means to work through network structures causes them to continue to seek and use traditional policies and management techniques that actually mitigate the positive attributes of networked arrangements [101]. Our findings support some aspects of study propositions 4 and 5 (see Table 1). Although data did not reveal a Network membership tension with respect to developing network style policy and practice, data did clearly support this phenomenon and tension with provincial governmental partners. They wanted to continue to construct policies and management techniques characteristic of traditional public service sectors; they sought infrastructure [61] and formalized organizational processes, such as formal collaborative structures, agreements, and governance procedures [65]. These efforts were indicative of an external macro-system contextual factor (i.e., provincial government partners) at play such that historical, social and political institutional forces encouraged growing formalization and centralization of the network, emulating traditional public service sectors [102].

The findings help to further explain the contextual influence of supportive policy environments [5] as well as politics and its history on early network formation [9] and on ongoing sustainability [86]. Centralized support, both fiscally and politically at multiple governmental levels, has been identified as a key factor in establishing cross-sectoral collaborations [5, 103]. In particular, access to initial funding as a catalyst for early Network formation as well as access to ongoing operational and ad hoc project funding for sustainability were critical factors [12, 86]. Given the similarities between the inter-organizational child health network studied here and ideas of collective impact and place-based approaches, these particular contextual factors deserve increased attention for their relevance in child health work.

The third contribution of this research concerns the notion of network-mediated system responsiveness. This is the first known health network study to identify a facilitating link between a health network and enhanced system responsiveness. These unanticipated consequences explain how the health network served as a catalyst or mediator in enhancing members’ abilities to respond individually and collectively to various contextual demands. Thus, perhaps the network may be seen as a driver of public system strengthening. This distinctive network-mediated multi-context responsiveness extends the traditional public service discourse of ‘responsiveness to client,’ with its narrow focus on agency or practitioner responsiveness to individual client needs [104]. Our findings revealed that the network took on collective issues that extended beyond their individual organizational mandates and traditionally defined clients groups. This extension beyond the individual child and the individual organization to cross-sectoral system responsiveness is also central to current place-based [50] and collective impact [84] approaches.

The concept of network responsiveness has not been identified in most germinal inter-organizational health network research [69]. One notable exception is Cunningham and colleagues [105] who assessed key factors related to the effectiveness and sustainability of clinical networks in Australia. Among several factors, they identified member participation and responsiveness within the network as short term measures of network effectiveness in one network.

Increased collaboration among network partners, which is an element of responsiveness, has been associated with network sustainability [7]. Sustaining the commitments among the involved partners, the capacity generated by the network, and the values generated from the partnership also have been associated with network sustainability [86]. Other study findings report unintended and unanticipated consequences of networks [106]. These may very well be elements of what we report as network *responsiveness*. Further, attempts to examine inter-organizational collaborations at multiple levels (i.e., micro: individual network partners, meso: across partner organizations, and macro: across the broader public system and communities) suggest that there is developing thinking around activity at multiple levels that feeds the network. There is much potential for networks, with their streamlined structures that are less formal than traditional government services, or some variation of them in certain circumstances, to be the driver of system responsiveness change.

Findings from this study need to be considered in light of study limitations. The reader should be careful to not generalize from a single case study design, but rather to consider the degree of theoretical transferability and fittingness to other contexts. The inability to account for participant memory selectivity and difficulties with past memory recall in the study methods is acknowledged. In some instances participants were asked to recall events from as far back as 13 years prior to data collection. A major strength of this work was the use of propositions as the theoretical basis for study design, allowing us to draw on many theoretical perspectives. The use of Framework Analysis methods, which was originally developed for public health system research purposes, strengthened the analytical process and the credibility of the findings.

### Future research

The study findings extend our current understanding of the powerful potential for this novel organizational type, inter-organizational child health networks, to positively shift the workings of the traditional public system. We reported on distinct forms of responsiveness at multiple levels. This responsiveness dimension may be an unrealized opportunity that requires particular attention in future research – the notion that a child health network (one that is often informally socially constructed and thus floats in a grey organizational zone) – might be more nimble and thus able to positively impact the responsiveness of the public system and the public system-third sector relationship space. The health (including social) policy and practice implications of this possibility are far reaching, and might serve to direct our attention to particular outcomes-based measurements associated with some inter-organizational health network impacts. This consideration opens new dialogue and the potential for future research to examine the role of health networks on system responsiveness, and ultimately, on individual and population health outcomes.

Future research ought to consider how child health networks have navigated traditional government policies and structures, and how government policy and processes have or have not shifted to accommodate health network organizational models. There is concern that these network types do not have a formal place in the legitimized public system, which has serious implications, politically and otherwise. Do these networks become part of the ‘hollow state’ [107] (i.e., as a metaphor for the use of third parties to deliver social services and act in the name of the state)? Are inter-organizational networks considered the ‘third sector’ (i.e., voluntary or not-for-profit sector) by government funders [108–110]? Are they ‘non-state providers’ [111] (i.e., small, often informal providers who are increasing in numbers, scope, scale and impact to fill the gap left by weak state capacity), even though many inter-organizational health networks are socially constructed to engage partners from multiple sectors, including governments and NGOs? Indeed, are such wicked problems [112–115] that health networks come together to tackle even governable [116]? Finally, what value can child health networks offer to place-based and collective impact approaches to child health work? Can child health networks be better designed to support these approaches so that leaders can align their efforts to tackle public system constraints to large scale social change?

## Conclusions

This study suggests that inter-organizational and cross-sectoral child health network leaders and their partners need to pay attention to relationship building within their network context, the political work of the network, and potential for network-mediated system responsiveness. These factors underlie the innovative and transformative nature of child health network practice. Thus, child health networks that attend to these processes can be dynamic, responsive, and perhaps more nimble in filling public system gaps that arise when formal services tackle tremendously complex issues within certain public and community-based system contexts.

## Abbreviations

AGM: Annual general meeting
NGO: nongovernmental organization

## Footnotes

1 This expression was used by the former Prime Minister of Canada, The Rt. Honourable Pierre Elliott Trudeau: “Living next to you is in some ways like sleeping with an elephant. No matter how friendly and even-tempered is the beast, if I can call it that, one is affected by every twitch and grunt” [117]. In the context of the child health network study, although there may be friendly relationships with this group, the size of the group (or their large funding contribution) has an impact on network activities or relationships.
